# Primary Extrapulmonary Rifampicin Mono-Resistant Tuberculosis (TB) of the Endometrium in a Sexually Inactive 20-Year-Old Indian Female: A Very Rare Case

**DOI:** 10.7759/cureus.32223

**Published:** 2022-12-05

**Authors:** Sankalp Yadav

**Affiliations:** 1 Medicine, Shri Madan Lal Khurana Chest Clinic, Moti Nagar, New Delhi, IND

**Keywords:** cartridge-based nucleic acid amplification test, multi-drug resistant tuberculosis, mycobacterium tuberculosis complex, female genital tract tuberculosis, tuberulosis

## Abstract

Female genital tract tuberculosis (TB) is less frequently reported, even in endemic countries. Often, the diagnosis is incidental and is seen with the involvement of pulmonary or extrapulmonary sites. Isolated female genital tract TB is quite rare, and cases of primary extrapulmonary rifampicin mono-resistant TB of the genital tract are rarest of the rare. In this case, a 20-year-old sexually inactive female with no history of TB presented with complaints of pain in her abdomen for past two months and absence of menstrual cycles since two years and was later diagnosed based on hysteroscopy, endometrial biopsy, and cartridge-based nucleic acid amplification test (CBNAAT) with primary extrapulmonary rifampicin mono-resistant TB of the endometrium. She was started on a WHO-recommended rifampicin mono-resistant TB regimen for 24 months but was ultimately lost to follow-up.

## Introduction

Tuberculosis (TB) is a leading cause of infection and, until the pandemic of COVID-19, was the highest contributor to morbidity and mortality in the world [1]. The disease is caused by the inhalation of *Mycobacterium tuberculosis* through aerosols generated by coughing, sneezing, spitting, or even speaking by an infected patient [2]. These aerosols range between 1-5 μm range and have the potential to infect others [3]. As per the latest WHO global TB report of 2022, approximately 10.6 million people grabbed the infection in the year 2021, with a majority, i.e., 45% cases reported from Southeast Asia [4].

The situation becomes grave with rise in the number of drug-resistant TB (DR-TB) cases [5]. In countries with a high burden of TB, it is a challenging task to timely detect and treat DR-TB cases [6]. This management becomes even more difficult when the disease is primary, i.e., with no history of TB and the focus of the infection lies in areas like the genital tract.

Herein, a case of primary extrapulmonary rifampicin mono-resistant TB of the endometrium in a sexually inactive 20-year-old female with no history of TB is presented. A detailed literature search revealed that no such case has ever been reported where the disease was localized to the endometrium with no pulmonary involvement for this age and sexual activity. Also, the primary rifampicin mono-resistance is never reported in female endometrium. Besides, this case was managed as per the national guidelines with the latest 24-month multidrug-resistant (MDR)-TB regimen recommended by the WHO in the year 2020.

## Case presentation

A 20-year-old unmarried female student belonging to a low socioeconomic group came as a referral case in the year 2020 with chief complaint of pain in the abdomen for two months and absence of menstrual cycles since two years. A detailed history of the present illness revealed that she had abdominal pain which was intermittent for two months. The pain was not associated with nausea, vomiting, or cramps and there were no aggravating or relieving factors. She also complained of the absence of menses for two years. There was a reduction in the flow for one year but for the last two years, there was a complete absence of menses. Her age of menarche was 12 years and the details of her menstrual cycles are given in Table 1. Further, there was no history of cough, weight loss, night sweats, and fever, and there was no history of TB in her or any of her contacts in the family.

**Table 1:** Menstrual history of patient NA: Not applicable

Year	Flow in days	Regular/Irregular	Pads per day	Frequency in days
2012-2015	3-4	Regular	4-6	26-28
2016	1	Irregular	1	28-30
2017-2019	0	NA	0	NA

She had consulted at nearby private clinics where she was given medroxyprogesterone acetate, which resulted in withdrawal bleeding. Further, the estrogen-progesterone challenge test with estradiol valerate and medroxyprogesterone acetate was suggestive of no withdrawal bleeding. Details of the treatment were unavailable.

General examination revealed a lean girl with a pulse of 70 per minute, blood pressure of 110/70 mmHg, respiratory rate of 17 per minute, a temperature of 98.4°F, weight 46 kg, body mass index (BMI) 16.9 kg/m^2^, and SpO_2_ of 97% on room air. She was pale but there was no edema, lymphadenopathy, cyanosis, or icterus. Her systemic examination was normal except for tenderness to touch in the hypogastric region. There was no swelling, organomegaly, or shifting dullness and the rest of the abdominal examination was within normal limits.

Pelvic examination revealed mucopurulent discharge with a normal cervix. A sample was taken from the cervix and sent for histopathological examination (HPE). The rest of per vaginal examination was normal.

A presumptive diagnosis of secondary amenorrhea under investigation was made with differentials as adnexal torsion, appendicitis, pelvic inflammatory disease (PID), urinary tract infection (UTI), pyelonephritis, kidney stones, and ovarian cysts. Routine tests were advised. Blood investigations were remarkable for hemoglobin 10.9 g/dl and erythrocyte sedimentation rate of 70 mm in the first hour. Urine for the pregnancy test was negative. Her HIV status was negative. The rest of her blood investigations were within normal range. A chest radiograph was normal.

**Figure 1:**
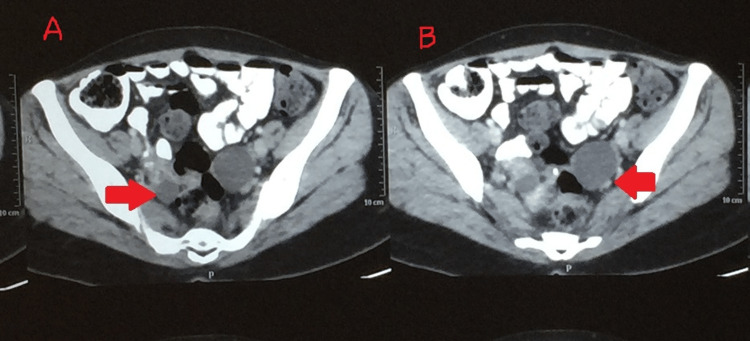
Multi-detector row computed tomography (MDCT) of the abdomen and pelvis showing bilateral cysts A) 30 mm follicular cyst in the right ovary (arrow); B) 37 mm X 31 mm cyst in the left ovary (arrow)

Induced sputum microscopy for acid-fast bacilli (AFB) and cartridge-based nucleic acid amplification test (CBNAAT) on induced sputum were negative. Hormonal assays of the follicle-stimulating hormone, luteinizing hormone, prolactin, and thyroid-stimulating hormone were unremarkable. Ultrasonography of the abdomen revealed a small-sized uterus (56 X 43 X 38 mm^3^) with a small amount of fluid in the endometrial cavity and a bilobed cyst with thin septa in the left ovary with a size of 52 mm X 28 mm. The right ovary was normal. A multi-detector row computed tomography (MDCT) of the abdomen and pelvis was suggestive of a 37 mm X 31 mm cyst with thin walls in the left ovary and a small 30 mm follicular cyst in the right ovary (Figure 1).

A hysteroscopy revealed shaggy, flimsy adhesions in the uterine cavity at the right cornual end. The left cornual end was also covered with adhesions. An endometrial biopsy sample was collected and sent for AFB smear, culture for *Mycobacterium tuberculosis*, and CBNAAT.

Reports of HPE of the cervix were negative. Endometrial biopsy revealed granulomatous endometritis with central caseation and few giant cells. CBNAAT of the tissue sample was positive for *Mycobacterium tuberculosis *(detected medium) with rifampicin resistance. Further, the culture was negative for any growth and drug susceptibility testing (DST) was not remarkable for any other resistance to second-line drugs.

Finally, a diagnosis of primary extrapulmonary rifampicin mono-resistant TB of the endometrium was made and the patient was planned for a WHO-recommended rifampicin mono-resistant TB regimen after a detailed pretreatment evaluation (PTE) as per the programmatic management of DR-TB (PMDT) guidelines [7]. Her PTE was within normal limits therefore according to her weight her regimen was decided as mentioned in Table 2.

**Table 2:** WHO-recommended rifampicin mono-resistant tuberculosis (TB) regimen in the year 2020

Drugs	Doses	Route of administration
Isoniazid	900 mg	Per oral
Ethambutol	1,200 mg	Per oral
Pyrazinamide	1,750 mg	Per oral
Moxifloxacin (high dose)	800 mg	Per oral
Ethionamide	750 mg	Per oral
Clofazimine	100 mg	Per oral
Kanamycin	750 mg	Intramuscular
Pyridoxine	100mg	Per oral

For the first two weeks, the patient tolerated the rifampicin mono-resistant TB regimen well with her symptoms of abdominal pain subsiding and she had no major adverse drug reactions. On her request, she was transferred out to her native district but was ultimately lost to follow-up. 

## Discussion

Genitourinary TB is an important extrapulmonary TB constituting 27% of all the reported cases in the world [8]. About 5-10% of infertile females are reported to have female genital tract TB globally [9]. In countries like India, Pakistan, and Bangladesh, where females are given less attention and/or are less comfortable in discussing about gynecological issues due to social stigma, several cases are not reported [10,11]. Besides, the misdiagnosis of abdominal pain and adnexal mass cases as ovarian tumors is also a substantial contributor to the no reporting/underreporting [12]. Female genital tract TB could present as genital ulcers, adnexal masses, and could mock many conditions like PID, dermoid, endometriosis, carcinoma, etc. [12]. The commonest sites of this type of TB are fallopian tubes (95-100%), the endometrium (50-60%), ovaries (20-30%), cervix (5-15%), myometrium (2.5%), and vagina/vulva (1%) [13,14].

Because of the paucibacillary nature and limited use of imaging methods like ultrasound, computerized axial tomography, MRI, and positron emission tomography to make a definite diagnosis of female genital tract TB, a very high degree of clinical qualm is essential during surgery when uncommon findings are noted like unexpected adhesions, caseous material, ascites, etc. [8,15].

MDR-TB is defined as TB where the strain of *Mycobacterium tuberculosis* is resistant to the two main antitubercular drugs, i.e., isoniazid and rifampicin [4,7]. Rifampicin mono-resistant TB is defined as a resistance to rifampicin without resistance to any other first‐line antitubercular drugs [4,7]. MDR-TB is a man-made problem and occurs either due to poorly designed treatment regimens, use of spurious/substandard drugs, improper dosages, and poor patient compliance due to lack of counseling or adverse drug reactions [4,5].

Some case reports and case series are available in the literature about female genital tract TB but reports of MDR-TB of the same are very rare and reports of primary rifampicin mono-resistant TB of the female genital tract with no history of TB have never been reported in a sexually inactive female.

A detailed study by Sharma et al. 2016, on six MDR-TB cases of the female genital tract, was the earliest report available in the literature [16]. However, their two cases were defined as cases of primary MDR-TB based on the location of the lesions i.e., in the female genital tract without evidence of TB elsewhere in the body [16]. They did not mention the past history of TB in these two cases which is extremely important to term a case of TB as a primary MDR-TB case. 

This present case is unique as there was no history of TB in the patient and the diagnosis was based on the endometrial biopsy which revealed granulomatous endometritis, hysteroscopy, and the CBNAAT of the tissue samples which were positive for *Mycobacterium tuberculosis* with rifampicin resistance. Besides, the two cases of Sharma et al., which were termed as primary MDR-TB of the female genital tracts, were in sexually active females of ages 28 and 30 years respectively [16]. But the subject of this present case was a sexually inactive 20-year-old unmarried female, thereby making it the first such presentation ever reported in the world.

The present case resembles the cases of Sharma et al. in the presence of abdominal pain and menstrual dysfunction [16]. All six cases of Sharma et al. had pallor and the same was noted in the present case as well [16]. However, there was only rifampicin resistance noted in the present case instead of both isoniazid and rifampicin resistance that was seen in all six cases with additional resistance to streptomycin and ethambutol resistance in some of their patients [16].

Moreover, the management of all six cases of Sharma et al. was as per the national protocol for the management of DR-TB with drugs kanamycin (intramuscular), levofloxacin, pyrazinamide, cycloserine, para-aminosalicylic acid, ethionamide, ethambutol, and pyridoxine, but the present case was given isoniazid, ethambutol, pyrazinamide, moxifloxacin (high dose), ethionamide, clofazimine, kanamycin, and pyridoxine as per the latest PMDT guidelines of the National Tuberculosis Elimination Programme (NTEP) present in the year 2020 [7,16].

This case should be used as an eye-opener for such rare instances where the primary focus was not in the lungs and the patient had no history of TB. Female genital tract TB could present as a number of clinical features like infertility (43-74%), oligomenorrhea (54%), abdominal pain (42.5%), amenorrhea (14%), dysmenorrhea (12-30%), menorrhagia (19%), dyspareunia (5-12%), and postmenopausal bleeding (2%) [12,13,17-19]. If left untreated, these have a propensity to significantly cause pelvic morbidity due to poor uterine receptivity, uterine adhesions, recurrent implantation failure, and infertility [12].

Also, the importance of the use of diagnostic techniques like the CBNAAT in large study populations for cases with abdominal pain and associated symptoms would help in the diagnosis of a higher number of cases that otherwise would have been missed. The only limitation of this case report was that the loop-mediated isothermal amplification could not be done due to the patient’s refusal.

To summarize, this was a case where a sexually inactive female with no history of TB was diagnosed as primary extrapulmonary rifampicin mono-resistant TB of the endometrium and was initiated on a WHO-recommended regimen for her weight after a detailed PTE as per the PMDT guidelines. The patient was followed-up initially when she showed improvements in her abdominal pain but ultimately was lost to follow-up most probably due to the stigma associated with the disease and/or issues related to overlooking of the female gender. 

## Conclusions

The present case is a very rare case with the reporting of primary rifampicin mono-resistant TB at an extrapulmonary site, i.e., endometrium in an unmarried, sexually inactive female. It is also remarkable that with the availability of investigations like CBNAAT the diagnosis was finally established when the culture was negative and the radiological techniques were inconclusive. The management was as per the WHO-recommended treatment regimen through the national TB elimination program.

To conclude, it is imperative to have a high index of suspicion for uncommon or never seen presentations of common diseases like TB. Further, the stigma associated with the female gender and other issues like misdiagnosis, underreporting, etc. need to be addressed for achieving the ultimate goal of TB elimination from India by 2025. 
